# GAT: a simulation framework for testing the association of genomic intervals

**DOI:** 10.1093/bioinformatics/btt343

**Published:** 2013-06-18

**Authors:** Andreas Heger, Caleb Webber, Martin Goodson, Chris P. Ponting, Gerton Lunter

**Affiliations:** ^1^MRC CGAT Programme and Functional Genomics Unit, ^2^MRC Functional Genomics Unit, Department of Physiology, Anatomy and Genetics, University of Oxford, Oxford OX1 3QX and ^3^University of Oxford, Wellcome Trust Center for Human Genetics, Roosevelt Drive, Oxford OX3 7BN, UK

## Abstract

**Motivation:** A common question in genomic analysis is whether two sets of genomic intervals overlap significantly. This question arises, for example, when interpreting ChIP-Seq or RNA-Seq data in functional terms. Because genome organization is complex, answering this question is non-trivial.

**Summary:** We present Genomic Association Test (GAT), a tool for estimating the significance of overlap between multiple sets of genomic intervals. GAT implements a null model that the two sets of intervals are placed independently of one another, but allows each set’s density to depend on external variables, for example, isochore structure or chromosome identity. GAT estimates statistical significance based on simulation and controls for multiple tests using the false discovery rate.

**Availability:** GAT’s source code, documentation and tutorials are available at http://code.google.com/p/genomic-association-tester.

**Contact:** andreas.heger@dpag.ox.ac.uk

## 1 INTRODUCTION

A common question in genomic analysis is whether two sets of genomic intervals, for example, ChIP-seq peaks and gene annotation classes, overlap significantly more than expected by chance alone. Interval overlap is easy to compute, but the significance can be computed analytically only for trivial situations. Hence, significance is usually estimated by simulation under some null model. This model must account for genome organization; a model that assumes independent and uniform placement of both interval sets is almost always inappropriate when testing for association with gene annotations because gene density strongly correlates with G + C content, and datasets of interest often also show G + C biases.

Here, we introduce Genomic Association Test (GAT), a tool for computing the significance of overlap between multiple sets of genomic intervals. GAT permits the restriction of the analysis to parts of a genome relevant to the experiment and accounts for chromosomal and isochore biases. Additional genomic features can be controlled for by providing additional segmentation files.

GAT’s approach was developed originally to test for the association of non-coding transcripts with other genomic elements (Ponjavic *et al.*, 2007), but has since been applied to a variety of problems, including:

- Conservation of non-coding transcription between human and mouse (Church *et al.*, 2009);
- Enrichment of histone marks and evolutionarily conserved genomic regions within non-coding transcripts (Marques and Ponting, 2009);
- Functional prediction of non-coding transcripts via their neighboring genes (Marques and Ponting, 2009); and
- Enrichment of ChIP-Seq binding events within signatures of open chromatin or disease-associated intervals (Ramagopalan *et al.*, 2010).

GAT's re-implementation delivers to the scientific community the extended functionality of the Ponjavic *et al.* (2007) methods.

## 2 USAGE

GAT is controlled from the command line. It requires at least three bed-formatted files that delimit genomic intervals (tuples of chromosome, start and end). The principal output of GAT is a table listing significant overlaps.

### 2.1 Input

Example: does a set of transcription factor binding site intervals from a ChIP-Seq experiment overlaps more than expected by chance with a set of DNaseI-hypersensitive sites? To perform this analysis, GAT requires three files:

1. A bed-formatted file with the intervals from the ChIP-Seq experiment *(Segments* ***S****)*. Several experiments can be supplied as multiple files or as a single file with multiple tracks.
2. A bed-formatted file with DNaseI-hypersensitive sites (*Annotations* ***A***). These could be obtained directly from the UCSC Genome Browser (Rosenbloom *et al.*, 2012). Several annotations from, for example, multiple cell lines can be supplied as multiple files or as a single file with multiple tracks.
3. A bed-formatted file with the workspace (***W***). The workspace defines the sequence that is accessible for the simulation. The simplest workspace contains the full genome assembly. In this example, the analysis should be restricted to only repeat-free regions, as only these are reliably mappable by short read data and thus could contain ChIP-Seq intervals. Again, appropriate bed-formatted files are available from the UCSC Genome Browser.

By default, the randomization procedure accounts for differences among chromosomes; for example, the X chromosome contains many sequence features that are atypical of autosomes. In addition to chromosome identity, local genomic G + C content is another common confounding factor. For example, G + C content might cause experimental biases in sequencing and hybridization protocols, while it is also a correlate of gene density (Lander *et al.*, 2001). To correct for G + C content, an optional bed-formatted file with the isochore structure of the genome can be supplied. GAT will then normalize by isochore and by chromosome. Here, isochores are discretized, for example, the genome is partitioned into windows falling into eight bins of different regional G + C content.

### 2.2 Output

In the aforementioned example, GAT will compute the overlap of ChIP-Seq binding events and DNaseI-hypersensitive sites. GAT will also estimate if the overlap is larger or smaller than expected by chance and will provide an empirical *P*-value of the statistical significance. If multiple ChIP-Seq experiments or multiple annotations have been submitted, GAT will compute the overlap for each combination of experiment and annotation and will estimate its significance. Storey's *q*-value (Storey and Tibshirani, 2003) or the Benjamini–Hochberg method (Benjamini and Hochberg, 1995) is used to control for multiple testing using a False discovery rate (FDR) procedure. 

## 3 IMPLEMENTATION

### 3.1 Overview

GAT is a python script (http://python.org) requiring only common and freely available numerical and scientific libraries. The memory and time-critical parts are implemented in cython (http://cython.org). It requires two collections of genomic intervals: *Segments* **(S)** and *Annotations* **(A)**. Each collection can contain one or more lists of genomic intervals (*S*_1_, *S*_2 _, *… , S_m_*; *A*_1_, *A*_2 _*, … , A_n_*). Intervals within a list of genomic intervals are required to be non-overlapping, and any overlapping intervals within **S** or **A** are merged prior to analysis. In addition, GAT requires a *Workspace* W describing the part of the genome accessible to the simulation. The analysis proceeds as follows. For each pair of interval lists *S_x_* and *A_y_* (*x ∈ *{1 , *… , m}, y ∈***{1 , *… , n*}), GAT computes the overlap between the intervals in S*_x_* and *A_y_* within workspace *W*: *observed* = |*S_x_*∩ *A_y_*∩ *W*|. |Here,| is the overlap operator and defaults to the number of nucleotides overlapping, but other operators (such as the number of segments) can be used. GAT subsequently creates randomly placed intervals in the genome with the same size distribution of *S_x_* within the workspace *W.* See below for simulation details. The overlap between each simulated set and *A_y_* is recorded. The average over all simulations represents the *expected* overlap. GAT reports the *fold enrichment* as the ratio of observed and expected overlap and associates an empirical *P*-value with it. GAT’s runtime and memory usage scale linearly with the number of simulations and the number and size of the genomic interval sets **S**, **A** and **W**.

### 3.2 Sampling method

The sampling method creates a list *R* of randomly placed intervals from an interval list *S_x_* within a workspace *W*. The sampling is done on a per-chromosome basis. For each chromosome *c*, randomly placed intervals are created by a two-step procedure:

1. Select an interval size from the empirical interval size distribution *S_x__,__c_*.
2. Select a position within the workspace *W_c_*.

Sampled intervals are added to *R_c_* until exactly the same number of nucleotides are in *R_c_* as are in *S_x__,__c_*. For reasons of performance, intervals are initially sampled without checking for overlap. Overlaps and overshoot are subsequently resolved in an iterative procedure once the sampled number of nucleotides approximates the target number.

The current sampling protocol is restricted to non-overlapping single segment intervals. Although amenable to many genomic features, it notably leaves discontinuous genomic segments, such as transcripts, untreated.

### 3.3 Isochores

Isochores are defined within GAT as chromosomal segments within a workspace. For each isochore *i*, the workspace W is subdivided into a workspace *W_i_* = *W *∩ *I_i_*. The sampling is performed separately for each *W_i_* and samples combined at the end. Isochores are thus treated in an equivalent manner to chromosomes. Isochores can be defined by G + C content, but can reflect any segmentation of the genome, such as chromatin marks.

## 4 CONCLUSIONS

GAT provides critical functionality for genomic analyses. By using standard BED files, it may be used alongside major data resources, such as the UCSC Genome Browser and Galaxy (Giardine *et al.*, 2005). GAT can be used in a similar context to GREAT (McLean *et al.*, 2010) and other tools, but can address a more diverse range of questions because of its simulation approach that takes into account both segment and annotation size distributions.

*Funding*: UK Medical Research Council.

*Conflict of Interest*: none declared.
